# Lower odds of remission among women with rheumatoid arthritis: A cohort study in the Swiss Clinical Quality Management cohort

**DOI:** 10.1371/journal.pone.0275026

**Published:** 2022-10-20

**Authors:** Enriqueta Vallejo-Yagüe, Julia N. Pfund, Theresa Burkard, Carole Clair, Raphael Micheroli, Burkhard Möller, Axel Finckh, Andrea M. Burden

**Affiliations:** 1 Department of Chemistry and Applied Biosciences, Institute of Pharmaceutical Sciences, ETH Zurich, Zurich, Switzerland; 2 Center for Primary Care and Public Health (Unisanté), University of Lausanne, Lausanne, Switzerland; 3 Department of Rheumatology, University Hospital Zurich, University of Zurich, Zurich, Switzerland; 4 Department of Rheumatology and Immunology, Inselspital, University Hospital of Bern, Bern, Switzerland; 5 Division of Rheumatology, University Hospitals of Geneva, Geneva, Switzerland; Nippon Medical School, JAPAN

## Abstract

**Objective:**

To compare the likelihood of achieving remission between men and women with rheumatoid arthritis (RA) after starting their first biologic or targeted synthetic disease-modifying anti-rheumatic drug (b/tsDMARD).

**Methods:**

This cohort study in the Swiss Clinical Quality Management in Rheumatic Diseases (SCQM) registry included RA patients starting their first b/tsDMARD (1997-31/04/2018). The odds of achieving remission at ≤12-months, defined by disease activity score 28-joints (DAS28) <2.6, were compared between men and women. Secondary analyses were adjusted for age and seropositivity, and we investigated potential mediators or factors that could explain the main findings.

**Results:**

The study included 2839 (76.3%) women and 883 (23.7%) men with RA. Compared to women, men were older at diagnosis and b/tsDMARD start, but had shorter time from diagnosis to b/tsDMARD (3.4 *versus* 5.0 years, p<0.001), and they had lower DAS28 at b/tsDMARD start. Compared to women, men had 21% increased odds of achieving DAS28-remission, with odds ratio (OR) 1.21, 95% confidence interval (CI) 1.02–1.42. Adjusting for age and seropositivity yielded similar findings (adjusted OR 1.24, 95%CI 1.05–1.46). Analyses of potential mediators suggested that the observed effect may be explained by the shorter disease duration and lower DAS28 at treatment initiation in men *versus* women.

**Conclusion:**

Men started b/tsDMARD earlier than women, particularly regarding disease duration and disease activity (DAS28), and had higher odds of reaching remission. This highlights the importance of early initiation of second line treatments, and suggests to target an earlier stage of disease in women to match the benefits observed in men.

## Introduction

Rheumatoid arthritis (RA), with a prevalence of 0.5 to 1.0% [1], affects women three times more frequently than men [2,3]. While the reason for this unbalanced frequency between women and men is not fully understood, it is known as the result from multiple factors, including both biologically-driven sex concepts and socially constructed gender factors. Sex-based factors include observed differences in the immune response and hormonal levels between sexes [4–7]. Comparing adult men and women, antibody production and cell-mediated immunity are generally enhanced in women [7,8]. Particularly, T cell activation and proliferation, CD4/CD8 ratio, B cells, and immunoglobulins are increased in women, while men present higher levels of CD8+ T cells and regulatory T (Treg) cells [7]. Additionally, levels of estrogen (which fluctuate during the menstrual cycle) have a dose-, density-, and distribute-dependent role in the immune-response [7], with low levels enhancing it, and high levels (characteristic of pregnancy stage) having immune inhibitory effect [8]. Conversely, progesterone and testosterone have anti-inflammatory effects [7,8]. Furthermore, besides the sex-based factors, socially constructed gender bias and stereotypes may also play a role in the development of RA by influencing environmental or behavioral factors [4]. For example, smoking, associated to increased risk of incidence of seropositive-RA [9], is more common among men than women in Switzerland [10]. And while women may seek health consultation earlier, they tend to be prescribed lower dose [11] or less appropriate pain medication than men [12–15]. Likewise, a later referral from first physician encounter to the arthritis clinic was reported for women *versus* men [16].

In addition to the differences in RA prevalence in men and women, previous studies have found differences in the presentation and management of RA. Among RA patients, an increased disease severity and higher pain levels in women, compared to men, have been reported in several studies [17–22]. Moreover, observational studies showed a stronger clinical response and higher likelihood of achieving remission in men compared to women [23–28]. However, these findings were not always consistent depending on the study design. For example, Jawaheer et al. found that men, compared to women, had higher odds of achieving sustained remission in early RA but not in established RA [24,26]. Likewise, Couderc et al. found inconsistent results on regard to remission rates and response across men and women depending on the follow-up time-point [29]. Additionally, a recent meta-analysis of randomized clinical trials (RCTs) for biologics reported no significant differences in response rates between women and men [30].

Thus, investigating the influence of sex and gender on the course of RA remains of interest. In this study we characterized women and men with RA at the time of starting their first biologic or targeted synthetic disease-modifying anti-rheumatic drug (b/tsDMARD), and aimed to assess the impact of sex/gender in the subsequent clinical response.

## Materials and methods

### Study design and data source

We conducted an observational cohort study using data from the Swiss Clinical Quality Management in Rheumatic Diseases (SCQM) registry. The SCQM registry, established in 1997, is a Swiss multicenter longitudinal cohort of patients with rheumatic diseases [31]. Patients are invited to enter the registry by their treating rheumatologist. Patients provide written informed consent, and they can withdraw their consent at any point. Following consent, information from routine visits is recorded [32]. Within these visits, clinical endpoints (e.g., inflammatory markers, disease activity score), patient-reported outcomes (e.g., pain), and treatments are recorded. Similarly, patient demographics, comorbidities, and other health-related notes are collected. Additional surveys are filled out by the patients regularly (e.g. Health Assessment Questionnaire (HAQ) [33]).

Pseudonymized data, without access to the code key, was provided by the Swiss Clinical Quality Management in Rheumatic Diseases (SCQM) registry to the researchers. Therefore, the commission of the Canton of Zurich (KEK: Req-2020-00045) waived the need for a full ethics authorization.

### Study population

The study included adult RA patients (≥18 years old) registered in the SCQM and who started their first b/tsDMARD between 1997 and April 30st 2018 (15-months before the end of the data collection period, July 31st 2019). The start of the first b/tsDMARD was considered the index date. Patients with a b/tsDMARD treatment start before their first visit registered in SCQM were excluded. Additionally, patients without a recorded visit at index date or within the previous 6-months (i.e., the baseline visit) were excluded.

### Exposure

The study exposure was the sex of the patients. While this is registered in the SCQM as female and male, we refer to these categories as women and men. Additionally, since both biologically-driven sex concepts and socially constructed gender factors may play a role in the study of this exposure groups, we refer to our exposure as sex/gender.

### Outcomes

The primary outcome was disease activity score 28-joints (DAS28) [33] remission within the first 12-months after the index date, defined as DAS28<2.6 [34]. DAS28-remission was calculated using erythrocyte sedimentation rate (ESR; DAS28-ESR). When DAS28-ESR was missing in follow-up visits, DAS28 using C-reactive protein (CRP; DAS28-CRP) was used instead, if available. Formulas for DAS28 are provided in **S1 Equations**.

Secondary outcomes were 1) the achievement of DAS28 low disease activity (LDA) or remission (DAS28-rem/LDA) within the first 12-months, defined as DAS28<3.2; 2) the achievement of Rheumatoid Arthritis Disease Activity Index-5 (RADAI-5) remission within the first 12-months, defined as RADAI-5 ≤1.4 [34].

For all outcomes, patients with missing records of the respective outcome during follow-up were considered as non-achievers of that outcome. To challenge this assumption, we performed a sensitivity analysis in which patients with missing follow-up information on the respective outcome were excluded.

### Follow-up

Patients were followed until achievement of the outcome, or for a maximum of 12-months. Sensitivity analyses that restricted and extended the maximum follow-up to 9- and 15-months, respectively, were performed.

### Covariates

Patients’ age at first symptoms and RA diagnosis were collected. Baseline patient characteristics were recorded at index date (start of first b/tsDMARD) or as close as possible to this date within a pre-defined look-back window. Patient demographics, body mass index (BMI), disease duration (time from RA diagnosis), rheumatoid factor (RF), anti-cyclic citrullinated peptide (anti-CCP) antibodies, number of swollen joint counts counting 28 (SJC28), number of tender joint counts counting 28 (TJC28), and physician global disease activity were collected with a 6-month look-back window. Inflammatory markers (ESR, CRP), disease activity scores (DAS28-ESR, DAS28-CRP, RADAI-5), and the Health Assessment Questionnaire (HAQ) [33] were collected with a 3-month look-back window. Information on treatment was collected at index date, including conventional synthetic disease-modifying anti-rheumatic drugs (csDMARD) and steroid use, and type of b/tsDMARD. Records on ever smoking, post-menopause, and comorbidities were collected if ever-before, except for fractures/surgeries/musculoskeletal system records and infections, which had a 6-month look-back window. Information on pregnancy or breast feeding had a 12-month look-back window.

RF and anti-CCP values were combined as seropositivity, which was positive if either was positive, negative if both were negative, and missing otherwise. Missingness in ever smoking, pregnancy/breast feeding, post-menopause record, and comorbidities, were categorized as absent.

### Statistical methods

Patient characteristics were described for women and men separately. To compare differences, findings in men were compared to those in women using chi-squared test for categorical variables, and t-test or Kruskal-Wallis test for continuous variables.

Multiple imputation with chained equations (MICE) was used to complete relevant baseline variables. We performed 55 imputations and the 41.2% of patients had complete information in every included variable. The variables included in the MICE, their role, their missingness, and the used imputed methods are detailed in **S1 Table**. Convergence was checked visually. Density plots depicting the overlapping of the distribution of key variables in the original and imputed datasets are presented in **S1 Fig**.

To assess the likelihood of achieving the study outcomes in men versus women (reference group), logistic regression was performed in each imputed dataset, and subsequently, results were combined using Rubin’s rule. These analyses were done crude and adjusted for age and seropositivity.

We identified as potential mediators or factors to explain an association between sex/gender and the study outcomes the following baseline covariates: DAS28, disease duration, BMI, and rheumatic medication. While mediators should not be included in the main model to assess the impact of an exposure on an outcome because they would disturb the findings, performing additional mediation analyses aids elucidating the pathways to explain the effect of an exposure on an outcome [35]. Thus, we performed mediation analyses to investigate the influence of the identified potential mediators on the impact of sex/gender on the achievement of DAS28-remission at ≤12-months. The above-mentioned potential mediators were treated as categorical variables, which were: moderate or high disease activity versus lower (DAS28≥3.2 versus DAS28<3.2); late versus early RA disease duration (RA duration >2-years versus ≤2-years); elevated BMI versus normal weight (BMI≥25 versus BMI<25); csDMARD use at index date (yes versus no); and steroid use at index date (yes versus no). Since adjusting for exposure-outcome confounders in mediation analyses may introduce additional biases [35], we did not adjust for exposure-outcome confounders.

The mediation analyses were performed independently for each potential mediator, and they included the following steps: I) Logistic regression to investigate the association between sex/gender and the potential mediator; II) Logistic regression to investigate the association between sex/gender and DAS28-remission when the potential mediator is included in the model [36]; Directed Acyclic Graphs (DAGs) showing the dependencies between the study exposure, outcome, and potential mediators were depicted in the **S2** and **S3 Figs**. When step-I showed no association between sex/gender and the potential mediator, further steps were not conducted. Finally, we concluded that there was a mediation effect when the findings from the respective mediation analysis showed an attenuated risk estimate compared to that from the main analysis [36].

Based on reviewer comments, we assessed the median change between follow-up and baseline (delta: follow-up value—baseline) for individual clinical endpoints and composite disease activity scores in a post-hoc analysis. For this analysis, only patients with information on the respective variables at both baseline and follow-up were included (complete case). When more than one measurement of a specific variable were available during follow-up (365 days) the best one was chosen.

All the analyses were performed with the R software, version 3.5.2. [37] MICE was conducted using the R package mice, version 3.13.0 [38].

### Ethics statement

This study was reviewed by the ethics commission of the Canton of Zurich (KEK: Req-2020-00045). Pseudonymized data, without access to the code key, was provided by the Swiss Clinical Quality Management in Rheumatic Diseases (SCQM) to the researchers. Therefore, the commission waived the need for a full ethics authorization.

## Results

**Fig 1:**
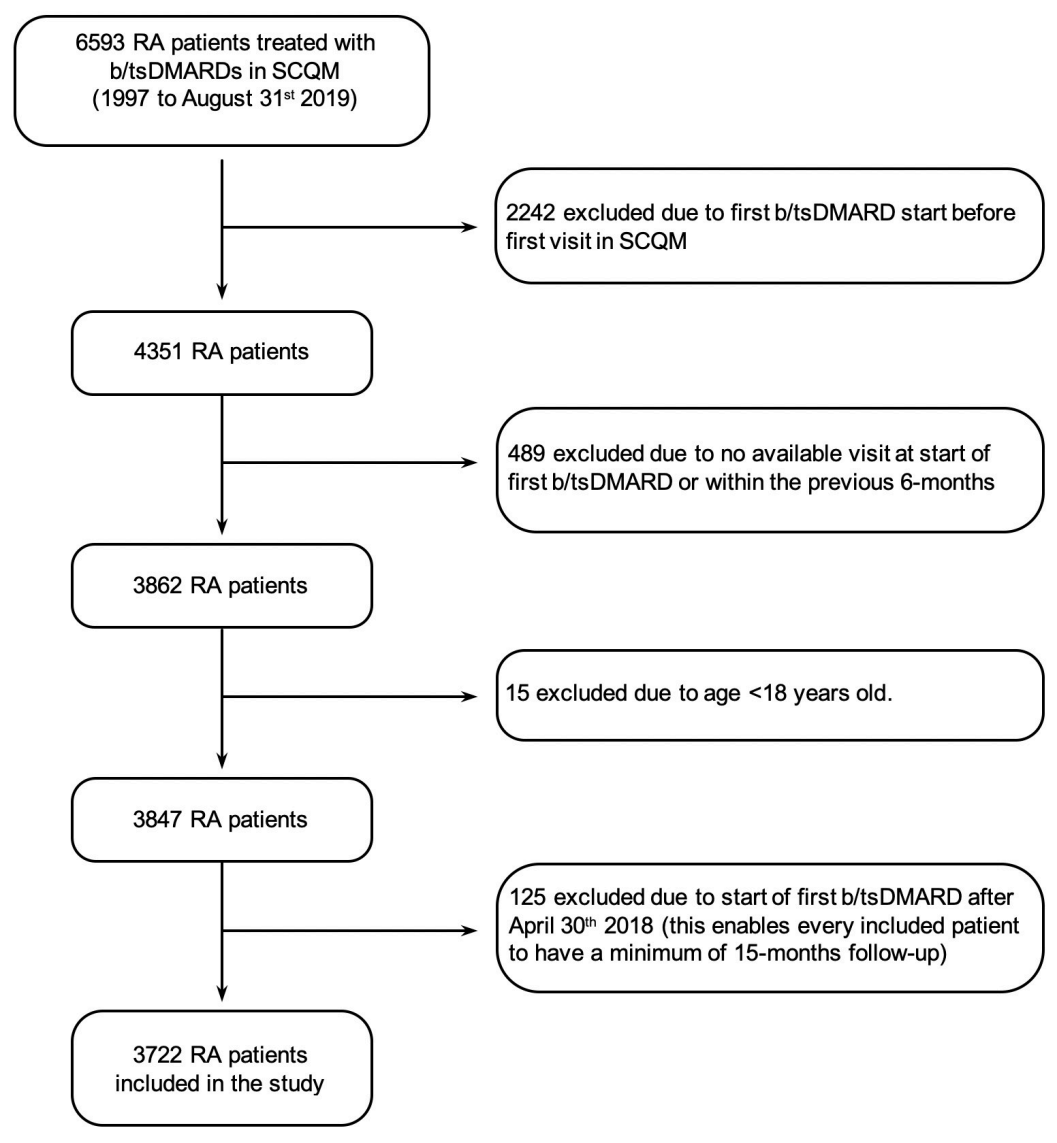
Flowchart of inclusion/exclusion criteria for the study population. Abbreviations: RA rheumatoid arthritis; b/tsDMARD biologic or targeted synthetic disease-modifying anti-rheumatic drug; SCQM Swiss Clinical Quality Management in Rheumatic Diseases.

The study included 3722 RA adult patients, of which 2839 (76.3%) were women and 883 (23.7%) were men. The flow chart for inclusion/exclusion criteria is depicted in **Fig 1**. Patient’s age at first symptoms and diagnosis, and baseline characteristics are presented in **Table 1**. Compared to women, men were older at first symptoms, diagnosis, and start of first b/tsDMARD. While both strata had similar time from first symptoms to diagnosis, men’s median time from diagnosis to the start of b/tsDMARD was shorter than the time in women (3.4 years versus 5.0 years). At baseline, men were more likely to be overweight (47.2% versus 27.0%) and a had higher frequency of ever smoking (49.2% versus 30.6%), compared to women. Conversely, men had a lower disease activity at baseline (DAS28-ESR 4.2 versus 4.4, p<0.001), and lower (better) median HAQ score (0.9 vs 1.0, p<0.001) and lower median TJC (4.0 vs. 5.0, p = 0.02), compared to women. When assessing patient responses to disease activity and pain level (on a scale from 1–10), we note that men had less reported pain (4.6 vs. 4.9, p = 0.05), and lower self-assessed activity of their rheumatic disease (4.8 vs. 5.1, p = 0.004) compared to women. Additionally, while men had higher frequency of hyperlipidemia, diabetes, and cardiovascular disease or cardiac events, women had significantly higher frequency of history of fibromyalgia, osteoporosis, and depression/anxiety.

**Table 1:** Patient characteristics.

	Women	Men	
	(n = 2839)	(n = 883)	*p*-value
**Patient characteristics at disease start**	** **	** **	** **
Age at first symptoms (mean (SD))	45.4 (15.0)	48.1 (14.0)	<0.001
missing (%)	105 (3.7)	28 (3.2)	
Age at diagnosis (mean (SD))	47.0 (14.8)	49.7 (13.6)	<0.001
missing (%)	89 (3.1)	32 (3.6)	
**Baseline patient characteristics (start of first b/tsDMARD)**	** **	** **	
Age at start of first b/tsDMARD, years (mean (SD))	55.4 (13.7)	56.5 (12.4)	0.029
Time from symptoms to diagnosis, years (median [IQR])	0.4 [0.1, 1.3]	0.3 [0.1, 1.0]	0.278
missing (%)	168 (5.9)	46 (5.2)	
RA duration, time from diagnosis to first b/tsDMARD, years (median [IQR])	5.0 [1.7, 12.0]	3.4 [1.2, 9.9]	<0.001
missing (%)	91 (3.2)	35 (4.0)	
BMI (kg/m^2^) (mean (SD))	25.2 (5.2)	26.5 (4.1)	<0.001
missing (%)	465 (16.4)	143 (16.2)	
BMI category (% from available)			<0.001
normal weight	1198 (50.5)	255 (34.5)	
overweight	642 (27.0)	349 (47.2)	
obese	404 (17.0)	127 (17.2)	
underweight	130 (5.5)	9 (1.2)	
Smoker ever before (%)	869 (30.6)	434 (49.2)	<0.001
Targeted treatment type (%)			0.499
TNF inhibitor biologic	2395 (84.4)	755 (85.5)	
other biologic	362 (12.8)	100 (11.3)	
tsDMARD	82 (2.9)	28 (3.2)	
Steroid use at index date (%)	1103 (38.9)	371 (42.0)	0.101
csDMARD use at index date (%)	1954 (68.8)	619 (70.1)	0.500
RF+ (%)	1957 (72.0)	607 (71.9)	0.986
missing (%)	122 (4.3)	39 (4.4)	
Anti-CCP+ (%)	1337 (64.3)	423 (65.3)	0.687
missing (%)	760 (26.8)	235 (26.6)	
ESR (median [IQR])	20.0 [10.0, 32.0]	18.0 [8.0, 34.0]	0.005
missing (%)	354 (12.5)	95 (10.8)	
CRP (mg/dL) (median [IQR])	0.8 [0.3, 1.4]	0.8 [0.4, 2.1]	0.001
missing (%)	1592 (56.1)	462 (52.3)	
Swollen joint count 28 (median [IQR])	6.0 [2.0, 10.0]	5.0 [2.0, 10.0]	0.603
missing (%)	49 (1.7)	15 (1.7)	
Tender joint count 28 (median [IQR])	5.0 [2.0, 11.0]	4.0 [1.0, 10.0]	0.020
missing (%)	54 (1.9)	21 (2.4)	
PRO Activity of rheumatic disease today (0–10) (mean (SD))	5.1 (2.6)	4.8 (2.7)	0.004
missing	549 (19.3)	175 (19.8)	
PRO Activity of rheumatic disease last 6 months (0–10) (mean (SD))	5.8 (2.5)	5.6 (2.5)	0.052
missing	570 (20.1)	178 (20.2)	
PRO How do you feel your health condition is today? (0–10) (mean (SD))	5.0 (2.5)	4.9 (2.5)	0.476
missing	574 (20.2)	175 (19.8)	
PRO Pain level today (0–10) (mean (SD))	4.9 (2.8)	4.6 (2.8)	0.047
missing	538 (19.0)	172 (19.5)	
Physician global disease activity (mean (SD))	4.8 (2.1)	4.8 (2.1)	0.883
missing (%)	1047 (36.9)	307 (34.8)	
DAS28-CRP (mean (SD))	4.1 (1.2)	4.1 (1.2)	0.471
missing (%)	1624 (57.2)	477 (54.0)	
DAS28-ESR (mean (SD))	4.4 (1.4)	4.2 (1.5)	<0.001
missing (%)	388 (13.7)	108 (12.2)	
RADAI-5 (mean (SD))	4.8 (2.1)	4.6 (2.2)	0.028
missing (%)	651 (22.9)	189 (21.4)	
HAQ (median [IQR])	1.0 [0.5, 1.6]	0.9 [0.4, 1.4]	<0.001
missing (%)	605 (21.3)	190 (21.5)	
Pregnancy/breast feeding	0 (0)	-	-
Post-menopause	896 (31.6)	-	-
Comorbidities			
Fibromyalgia	54 (1.9)	4 (0.5)	0.004
Osteoporosis	601 (21.2)	111 (12.6)	<0.001
Other rheumatological disease	773 (27.2)	219 (24.8)	0.167
Hyperlipidemia	136 (4.8)	80 (9.1)	<0.001
Diabetes	133 (4.7)	89 (10.1)	<0.001
Cardiovascular disease / cardiac event	886 (31.2)	340 (38.5)	<0.001
Cancer	77 (2.7)	21 (2.4)	0.674
Depression/anxiety	353 (12.4)	66 (7.5)	<0.001
Fractures, surgeries, musculoskeletal system	240 (8.5)	60 (6.8)	0.131
Infections	41 (1.4)	16 (1.8)	0.535
Latent inactive tuberculosis	46 (1.6)	16 (1.8)	0.812

Values are the counts and percentages unless stated otherwise. Abbreviations: b/tsDMARD biologic or targeted synthetic disease-modifying anti-rheumatic drug; RA rheumatoid arthritis; BMI body mass index; TNF tumour necrosis factor; tsDMARD targeted synthetic disease-modifying anti-rheumatic drug; csDMARD conventional synthetic disease-modifying anti-rheumatic drug; RF rheumatoid factor; Anti-CCP anti cyclic citrullinated peptide; ESR erythrocyte sedimentation rate; CRP C-reactive protein; PRO patient reporting outcome; DAS28 disease activity score 28; RADAI-5 rheumatoid arthritis disease activity index-5; HAQ health assessment questionnaire.

**Fig 2:**
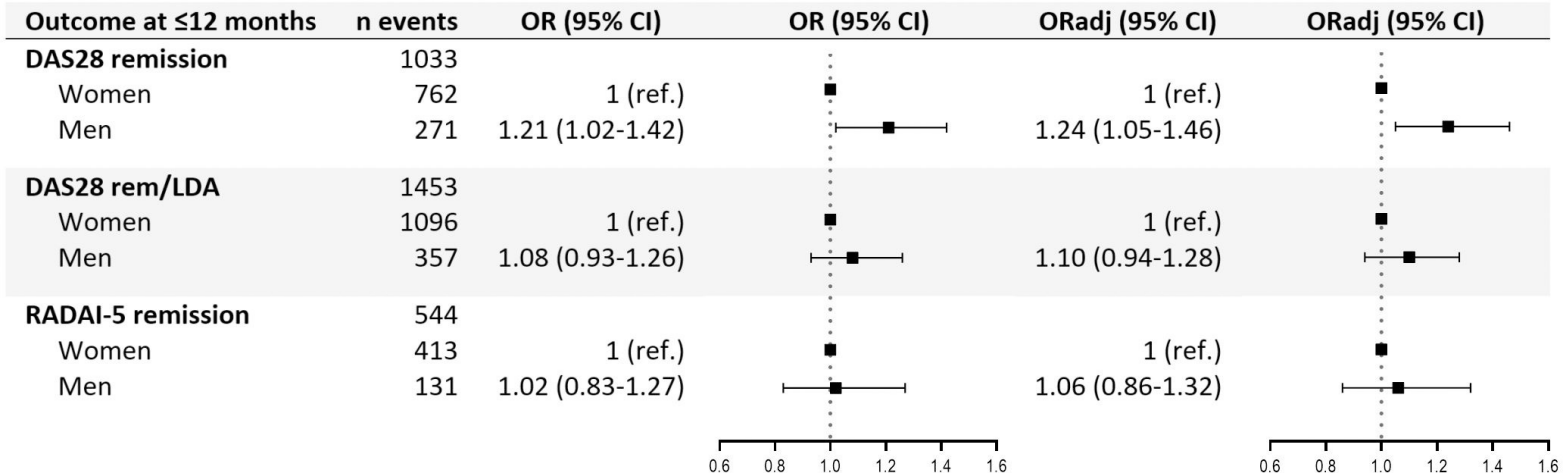
Logistic regression assessing the effect of sex/gender on the study outcomes, with 12-month maximum follow-up. Abbreviations: n number; OR odds ratio; ORadj odds ratio adjusted for age and seropositivity; DAS28 Disease Activity Score 28; rem/LDA remission or low disease activity; RADAI-5 Rheumatoid Arthritis Disease Activity Index-5.

Results from the main logistic regressions are presented in **Fig 2**. Compared to women, men had higher odds of achieving DAS28-remission within the first 12-months, with unadjusted odds ratio (OR) of 1.21 and 95% confidence interval (CI) 1.02–1.42. Adjusting for age and seropositivity led to similar results, with adjusted OR (ORadj) 1.24 (95% CI 1.05–1.46). Secondary outcomes DAS28-rem/LDA and RADAI-5 remission did not show a significant difference between men and women. Sensitivity analyses with 9- and 15-months follow-up showed similar results as those at 12-months for all three outcomes (**Table 2**).

**Table 2:** Sensitivity analysis. Logistic regression assessing the effect of sex/gender on the study outcomes, with maximum follow-up of 9- and 15-months.

	Outcome at ≤9-months			Outcome at ≤15-months		
Outcome	n events	OR (95% CI)	ORadj (95% CI)	n events	OR (95% CI)	ORadj (95% CI)
**DAS28 remission**	613			1208		
Women	447	1 (ref.)	1 (ref.)	888	1 (ref.)	1 (ref.)
Men	166	1.24 (1.02–1.51)	1.24 (1.02–1.51)	320	1.25 (1.07–1.46)	1.28 (1.09–1.50)
**DAS28 rem/LDA**	868			1680		
Women	653	1 (ref.)	1 (ref.)	1268	1 (ref.)	1 (ref.)
Men	215	1.08 (0.90–1.29)	1.09 (0.91–1.30)	412	1.08 (0.93–1.26)	1.10 (0.95–1.28)
**RADAI-5 remission**	281			672		
Women	218	1 (ref.)	1 (ref.)	507	1 (ref.)	1 (ref.)
Men	63	0.92 (0.69–1.24)	0.95 (0.71–1.27)	165	1.06 (0.87–1.28)	1.09 (0.90–1.33)

Abbreviations: n number; OR odds ratio; ORadj odds ratio adjusted for age and seropositivity; DAS28 Disease Activity Score 28; rem/LDA remission or low disease activity; RADAI-5 Rheumatoid Arthritis Disease Activity Index-5.

Sensitivity analysis excluding patients without follow-up information on the outcome (**S2 Table**), showed an even higher likelihood of men versus women on achievement of DAS28-remission within the first year (OR 1.36 [95% CI 1.12–1.64]). Likewise, this resulted in higher odds of men achieving DAS28-rem/LDA (OR 1.23 [95% CI 1.02–1.50]), whereas the findings for RADAI-5 remission remained non-significant.

Results from the mediation analyses are shown in **Table 3**, and **S3 Fig**. Among the investigated potential mediators, men were associated with lower odds of baseline DAS28≥3.2 and RA duration >2-years, and higher odds of having BMI≥25 in comparison to women. Subsequently, adding baseline DAS28 (threshold 3.2) to the main analysis removed the significance from the previously observed association between sex/gender and DAS28-remission. Similar observation was made for RA duration (threshold 2-years). Thus, baseline DAS28 and RA duration were identified as factors which may explain the association between sex/gender and DAS28-remisison at ≤12-months. Conversely, adding BMI to the main analysis did not attenuate, nor dismissed, the main findings.

**Table 3:** Summary findings from the mediation analyses (steps I and II), and findings from the main analysis for reference.

	Main analysis		Mediation analyses	
			Step-I	Step-II
	E -> O		E -> M	E + M -> O
	OR (95%CI)	ORadj (95%CI)	OR (95%CI)	OR (95%CI)
**Mediation analysis for baseline DAS28≥3.2**				
Female	1 (ref.)	1 (ref.)	1 (ref.)	1 (ref.)
Male	1.21 (1.02–1.42)	1.24 (1.05–1.46)	0.65 (0.53–0.78)	1.15 (0.96–1.38)
DAS28<3.2	-	-	-	1 (ref.)
DAS28≥3.2	-	-	-	0.36 (0.30–0.43)
**Mediation analysis for RA disease duration at index date >2years**				
Female	1 (ref.)	1 (ref.)	1 (ref.)	1 (ref.)
Male	1.21 (1.02–1.42)	1.24 (1.05–1.46)	0.71 (0.60–0.83)	1.17 (0.99–1.39)
RA≤2years	-	-	-	1 (ref.)
RA>2years	-	-	-	0.74 (0.64–0.87)
**Mediation analysis for baseline BMI≥25**				
Female	1 (ref.)	1 (ref.)	1 (ref.)	1 (ref.)
Male	1.21 (1.02–1.42)	1.24 (1.05–1.46)	2.29 (1.93–2.72)	1.31 (1.09–1.58)
BMI<25	-	-	-	1 (ref.)
BMI≥25	-	-	-	0.73 (0.62–0.86)
**Mediation analysis for csDMARD use at index**				
Female	1 (ref.)	1 (ref.)	1 (ref.)	-
Male	1.21 (1.02–1.42)	1.24 (1.05–1.46)	1.06 (0.90–1.25)	-
**Mediation analysis for steroid use at index**				
Female	1 (ref.)	1 (ref.)	1 (ref.)	-
Male	1.21 (1.02–1.42)	1.24 (1.05–1.46)	1.14 (0.98–1.33)	-

Abbreviations: E study exposure (sex/gender); O study outcome (DAS28-remission at ≤12-months); M potential mediator; DAS28 Disease Activity Score 28; OR Odds ratio; ORadj odds ratio adjusted for age and seropositivity; csDMARD conventional synthetic disease-modifying anti-rheumatic drugs.

Results from the post-hoc analysis assessing changes in individual clinical endpoints and composite disease activity scores are provided in **S3 Table**. We did not identify any significant differences, apart from CRP, which had substantial missingness.

## Discussion

This study on 3722 RA patients new-users of b/tsDMARD had 76% women and 24% men. At baseline, men were older and presented higher frequency of overweight and ever smoking compared to women. Conversely, women had longer disease duration (5.0 versus 3.4 years in men), more active disease (DAS28, RADAI-5), and more frequent history of fibromyalgia, osteoporosis, and depression/anxiety. Men were more likely to achieve DAS28-remission within one year, in comparison to women. Our analyses suggested that this benefit in men may be explained by the differences in baseline disease activity (DAS28) and disease duration at start of first b/tsDMARD. Conversely, no differences were observed between men and women on their likelihood of achieving low disease activity (DAS28-rem/LDA) or RADAI-5 remission.

Results from our analyses showed 21% higher odds of DAS28-remission in men versus women, which was consistent with previous studies. For example, an observational cohort study with 2879 RA patients treated with tumor necrosis factor alpha inhibitor (TNFi) (etanercept/infliximab) in a British registry suggested that women were 40% significantly less likely to achieve DAS28-remission [27]. Similarly, another study in the same registry, seeking to identify predictors for infliximab response at 12-months, reported women to be significantly associated with lower DAS28 improvement compared to men [28]. Conversely, two studies of RA patients treated with rituximab and abatacept, respectively, showed no significant differences between men and women and their likelihood of achieving DAS28-remission [29,39]. However, these last-mentioned studies included in their main analyses adjustment for baseline disease activity and disease duration. While the authors treated these variables as confounders, we identified them as mediators, and adjustment for mediators in their analyses is expected to attenuate or remove the observed effect of the study exposure. Additionally, findings from the rituximab study showed higher remission rates in men compared to women [29], and the un-adjusted results from the abatacept study showed significantly reduced odds of achieving DAS28-remission in women compared to men at 6-, 12-, and 24-months, with an OR 0.56 (95% CI 0.37 to 0.87) in women at 12-months [39].

Similarly, a recent meta-analysis of RCTs of biologics did not find statistically significant difference in the American College of Rheumatology 20 (ACR20) rates between men and women. While both DAS28-remisson and ACR20 are commonly used clinical outcomes in RA, they are different in nature. The ACR20 is a relative score, defined by a 20% improvement in various disease attributes (e.g. SJC28, TJC28, pain, HAQ, CRP) [34]. Thus, ACR20 outcome is taking the baseline disease activity and HAQ into consideration. This, together with the above-mentioned role of the baseline disease activity as mediator in the association between sex/gender and clinical outcome, may explain why no differences were found between men and women when using ACR20 as outcome.

Our mediation analyses suggested baseline disease activity and disease duration as mediators of the effect of sex/gender on the achievement of DAS28-remisison. More concretely, the observed lower odds of having moderate or higher disease activity at baseline (DAS28≥3.2) and lower odds of a disease duration >2-years at start of b/tsDMARD explained the observed association between men and higher odds of achieving DAS28-remissison compared to women.

In our study, men were prescribed b/tsDMARD with a median 1.6 years shorter disease duration than women. This significant temporal gap between men and women escalating treatment was consistent with other observational studies on RA patients treated with biologics [29,39,40].

The study by Arkema et al. in the Swedish national biologics registry ARTIS suggested that, compared to men, women started TNFi therapy at a higher level of patient-reported outcomes (TJC28, patient’s global assessment, pain, HAQ) but at similar level of physician-reported outcomes (SJC28, physician’s global assessment) [40]. Our study reflected a similar trend, with women having worse symptoms or patient-reported outcomes (TJC28, RADAI-5), while similar physician-reported outcomes (SJC28, physician global disease activity), in comparison to men at baseline.

Thus, although overall decision on step-up to b/tsDMARD in the Swiss practice is done based on disease activity, we observed discrepancies on composite disease activity scores (DAS28, RADAI-5) and individual clinical features between the study groups. Hypotheses to explain these phenomena may include differential physician-driven and/or patient-driven aspects on treatment decision-making.

Although patient-reported outcomes, like pain or tenderness, are key features on RA disease management, sex-driven differences (e.g., hormonal level; expression of pain receptors) and socially-constructed gender stereotypes may affect their perception, expression, and interpretation across patients [41]. Studies on sex, gender, and pain reported that, while chronic pain syndromes are more frequent in women, and women generally report more and worse pain than men, there are numerous examples of women’s pain being discounted or underplayed in healthcare [12,13,42–46], and women often receive less or less appropriate pain medication [12–15]. While our findings show similar type of b/tsDMARD and similar frequency of comedication with csDMARD and steroids between men and women, the shorter disease duration and lower disease activity in men at b/tsDMARD start, compared to women, reflects a potential treatment gap between the study groups. Additionally, in the context of pain, we may discuss depression, as a common comorbidity with pain [14] and fibromyalgia. In our study, the female/male ratio for fibromyalgia was four to one (1.9% of women versus 0.5% of men), and frequency of depression/anxiety was 12.4% in women and 7.5% in men. While overall these frequencies are low, this along with the observed higher tender joints in women, suggest that women may have had higher pain at the start of their first b/tsDMARD, in comparison to men. And yet, it may be that a more frequent mention to pain by women may have led physicians to unconsciously “raise the bar” and undervalue the patient’s observations, a move that by itself may “increase the voice” of the patient, all in all turning into a vicious circle from which healthcare professionals should seek to escape.

In addition to the above-discussion on patient-reported outcomes, we may as well discuss the levels of inflammatory markers in both study groups. Conversely to the study by Arkema et al. [40], we observed higher ESR in women versus men at baseline, whereas in both theirs and our study, baseline CRP was higher in men. Both ESR and CRP are acute phase reactants commonly used in the clinic as biomarkers of inflammation. Both increase with age [47], may be differently affected by sex and BMI [48], and have different half-life [49]. It was suggested that ESR is more influenced by sex (elevated in females) and age, while the CRP levels may be affected by smoking, high blood pressure, and high BMI [50]. This could partially explain our higher baseline CRP in the male population, with higher BMI and prevalence of cardiovascular history and smoking. Likewise, it could partially explain the observed higher baseline ESR level in women. While evidenced interpretation of the controversy between the baseline findings on ESR and CRP remain unclear, our CRP results may be taken with caution due to the high missingness (>50% at baseline), and the elevated ESR levels (which agreed with the elevated tender joint counts and RADAI-5 score in women) depicted a more active disease in women *versus* men at start of treatment.

It remains of interest to investigate the impact of sex on the b/tsDMARD mode of action. RA clinical guidelines do not differentiate treatment regimen based on sex [41], and although further sex-specific pharmacological research remains of interest, this lays out of the scope and capacity of our study design. However, independently of sex-specific mechanisms, according to our study findings, a higher threshold for prescription of b/tsDMARD in women *versus* men may partially explain the observed gap on treatment. While this could be prescriber-driven, we also note that observations from our practice in Switzerland also describe women patients as more reluctant to b/tsDMARDs and prescription of injectable therapies compared to men.

Ultimately, the study findings suggest that earlier step-up treatment with b/tsDMARD in women could potentially increase their odds of remission and it is consistent with the suggestions that earlier treatment with biologics leads to higher likelihood of successful clinical outcome [51].

Results for our secondary outcome DAS28-rem/LDA showed no differences across men and women. While this could simply be due to the more approachable threshold of DAS28-rem/LDA compared to DAS28-remisison, exclusion of the patients without follow-up information on outcome (sensitivity analysis) tilted the odds towards a favorable result for men. This suggests a differential distribution of patients lacking follow-up information on outcome between sexes. Therefore, the study findings on DAS28-rem/LDA should be taken with caution. On regard to the outcome RADAI-5 remission, men and women had similar odds of achieving RADAI-5 remission in both the main and the sensitivity analysis. RADAI-5 is a mainly patient-driven score, and therefore, it reflects the patient’s perception of their own disease, unlike the DAS28, which mostly contains values measured by the physicians (3-item formula for DAS28, S1 Equations). Thus, these two outcomes measured two different clinical goals. A complete understanding on the RADAI-5 results remains of interest.

### Strengths and limitations

Key strengths of this study include the large sample size and the addition of the mediation analyses, which aid the discussion and understanding of the findings. An additional strength is the appropriateness of the SCQM data to address the research question. While missing values are always a burden in real-world-data, key covariates were sufficiently complete so that their missingness could be successfully addressed by MICE. The decision to consider patients missing follow-up information on outcome as not outcome achievers was tested with a sensitivity analysis, which supported the study findings on DAS28-remission. We would have excluded patients in remission at baseline, however, to avoid having different sample size in each imputed dataset, we decided against it. We did not quantify the effect of the mediation; thus, it may be that the two identified mediators fully explain the observed association between sex/gender and remission, or there could be other mediators that we did not foresaw. For example, we did not explore the role of depression or fibromyalgia in the mediation analyses. And we acknowledge that the comorbidities (e.g., fibromyalgia) may be underreported.

Although we would have liked to investigate DAS28-CRP remission in parallel to the studied DAS28-ESR remission, we were limited by the high missingness in CRP. Additionally, although a recent study in the Veterans Affairs RA (VARA) registry (10.2% women) suggested that their observed lower likelihood of achieving DAS28-ESR remission in women *versus* men was driven by ESR and that ESR could be biased in sex studies due to the influence of sex on ESR [52]; the rheumatology guidelines do not distinguish between DAS28-ESR and DAS28-CRP [48]. Moreover, the threshold currently used for DAS28 remission was created and validated using DAS28-ESR [50]. Thus, since the real-world RA population has 70% women (conversely to the VARA registry), it is expected that DAS28-ESR remission remains an optimal measure for our research question.

While we acknowledge a gender concept beyond the binary, due to data limitations we were restricted to the study of women and men as a binary category. Additionally, when discussing the study results in the context of other findings, since the concepts sex and gender were often interchanged in biomedical literature [53], and the terms women/men and female/male were indistinctly used, we compared other studies’ women and female patients with our women cohort, and men and male patients with our men cohort.

Finally, following the sex differences in the immune function and the effects of sex hormones on the immune response, described elsewhere [53–55], a limitation of this study was the absence of sex hormonal data. Additionally, studying the effects of pregnancy, post-partum, and menstrual cycles on the study outcome in women (or people with cycles) remains of interest. For example, it has been suggested that the risk of RA could be reduced during pregnancy [55], however, our study population did not include any patient with record of pregnancy (nor breast feeding) at baseline.

## Conclusion

The study findings indicated that when starting their first b/tsDMARD, men had a shorter disease duration and lower disease activity (DAS28-ESR, RADAI) in comparison to women. Likewise, compared to women, men presented higher odds of achieving DAS28-remission within the first year. Finally, findings from mediation analyses indicate these discrepancies in the treatment decision making regarding start of first b/tsDMARD in men and women patients as potential explanation for the observed response gap between the sex groups. Thus, these findings suggest that step-up to b/tsDMARD treatment at an earlier disease stage in women, similar to the observed practice in men, may bring women the beneficial effect observed in the men group.

## Supporting information

S1 Fig **Density plots depicting the distribution of key variables in the original dataset (blue) and the imputed datasets (red).** Abbreviations: BMI body mass index; RA_duration rheumatoid arthritis duration; esr erythrocyte sedimentation rate; n_swollen_joints_28 number of swollen joints counting 28; n_teder_joints_28 number of tender joints counting 28; radai_5_score Rheumatoid Arthritis Disease Activity Index-5; DAS28_score Disease Activity Score 28.
(PDF)

S2 Fig Directed acyclic graphs (DAGs) showing the dependencies between the study exposure sex/gender (E; blue balloons), the study outcome DAS28-remission (O; green balloons), and the covariates age and seropositivity (grey balloons). Each DAG is accompanied by the respective odds ratio (OR) or adjusted OR (ORadj) with 95% confidence interval (CI). Abbreviations: DAS28 Disease Activity Score 28; sero+ seropositivity.
(PDF)

S3 Fig Mediation analyses. Directed acyclic graphs (DAGs) showing the dependencies between the study exposure sex/gender (E; blue balloons), the study outcome DAS28-remission (O; green balloons), and the potential mediators (M; brown balloons), for the association between sex/gender and DAS28-remission. Each DAG is accompanied by the respective odds ratio (OR) with 95% confidence interval (CI). Abbreviations: DAS28 Disease Activity Score 28; RA rheumatoid arthritis; BMI body mass index; csDMARD conventional synthetic disease-modifying anti-rheumatic drug; E exposure; O outcome; M mediator.
(PDF)

S1 Table Details about the variables and methods used in the multiple imputations by chain equations (MICE). The 41.2% of the patients had complete information on every variable included in the MICE.
(PDF)

S2 Table Sensitivity analysis, excluding patients without any record on the outcome information during follow-up. Logistic regression assessing the effect of sex/gender on the study outcomes, with maximum follow-up of 12-months.
(PDF)

S3 Table Post-hoc analysis. Changes in individual clinical endpoints and composite disease activity scores (delta: Follow-up value—baseline). As a note, median values below zero reflect improvement, reduction of the scores.
(PDF)

S1 Equations (PDF)
