# Supplementary material for: Lower odds of remission among women with rheumatoid arthritis: A cohort study in the Swiss Clinical Quality Management cohort
Source: PLoS One. 2022 Oct 20;17(10):e0275026. doi: 10.1371/journal.pone.0275026 (PMC9584448; doi:10.1371/journal.pone.0275026)
Supplement: S1 Fig — Density plots depicting the distribution of key variables in the original dataset (blue) and the imputed datasets (red). Abbreviations: BMI body mass index; RA_duration rheumatoid arthritis duration; esr erythrocyte sedimentation rate; n_swollen_joints_28 number of swollen joints counting 28; n_teder_joints_28 number of tender joints counting 28; radai_5_score Rheumatoid Arthritis Disease Activity Index-5; DAS28_score Disease Activity Score 28. (PDF) [file pone.0275026.s001.pdf]

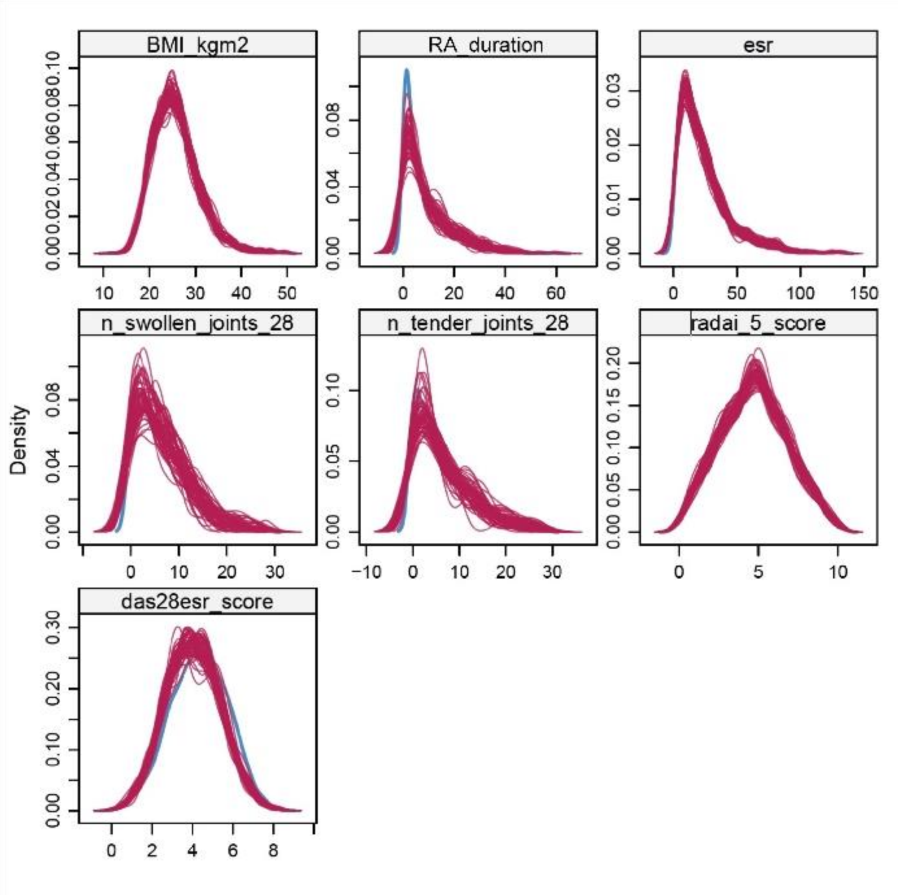

S3 Fig. Density plots depicting the distribution of key variables in the original dataset (blue) and the imputed datasets (red). Abbreviations: BMI body mass index; RA_duration rheumatoid arthritis duration; esr erythrocyte sedimentation rate; n_swollen_joints_28 number of swollen joints counting 28; n_teder_joints_28 number of tender joints counting 28; radai_5_score Rheumatoid Arthritis Disease Activity Index-5; DAS28_score Disease Activity Score 28.
