# Supplementary material for: Lower odds of remission among women with rheumatoid arthritis: A cohort study in the Swiss Clinical Quality Management cohort
Source: PLoS One. 2022 Oct 20;17(10):e0275026. doi: 10.1371/journal.pone.0275026 (PMC9584448; doi:10.1371/journal.pone.0275026)
Supplement: S2 Fig — Each DAG is accompanied by the respective odds ratio (OR) or adjusted OR (ORadj) with 95% confidence interval (CI). Abbreviations: DAS28 Disease Activity Score 28; sero+ seropositivity. (PDF) [file pone.0275026.s002.pdf]

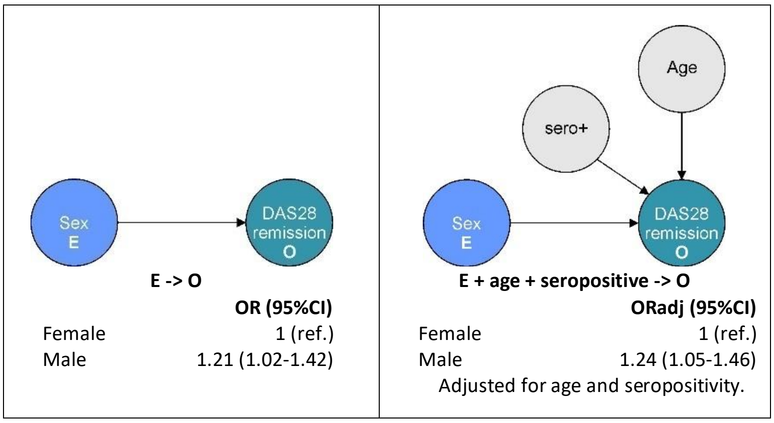

S4 Fig. Directed acyclic graphs (DAGs) showing the dependencies between the study exposure sex/gender (E; blue balloons), the study outcome DAS28-remission (O; green balloons), and the covariates age and seropositivity (grey balloons). Each DAG is accompanied by the respective odds ratio (OR) or adjusted OR (ORadj) with 95% confidence interval (CI). Abbreviations: DAS28 Disease Activity Score 28; sero+ seropositivity.
