# Supplementary material for: Lower odds of remission among women with rheumatoid arthritis: A cohort study in the Swiss Clinical Quality Management cohort
Source: PLoS One. 2022 Oct 20;17(10):e0275026. doi: 10.1371/journal.pone.0275026 (PMC9584448; doi:10.1371/journal.pone.0275026)
Supplement: S3 Fig — Directed acyclic graphs (DAGs) showing the dependencies between the study exposure sex/gender (E; blue balloons), the study outcome DAS28-remission (O; green balloons), and the potential mediators (M; brown balloons), for the association between sex/gender and DAS28-remission. Each DAG is accompanied by the respective odds ratio (OR) with 95% confidence interval (CI). Abbreviations: DAS28 Disease Activity Score 28; RA rheumatoid arthritis; BMI body mass index; csDMARD conventional synthetic disease-modifying anti-rheumatic drug; E exposure; O outcome; M mediator. (PDF) [file pone.0275026.s003.pdf]

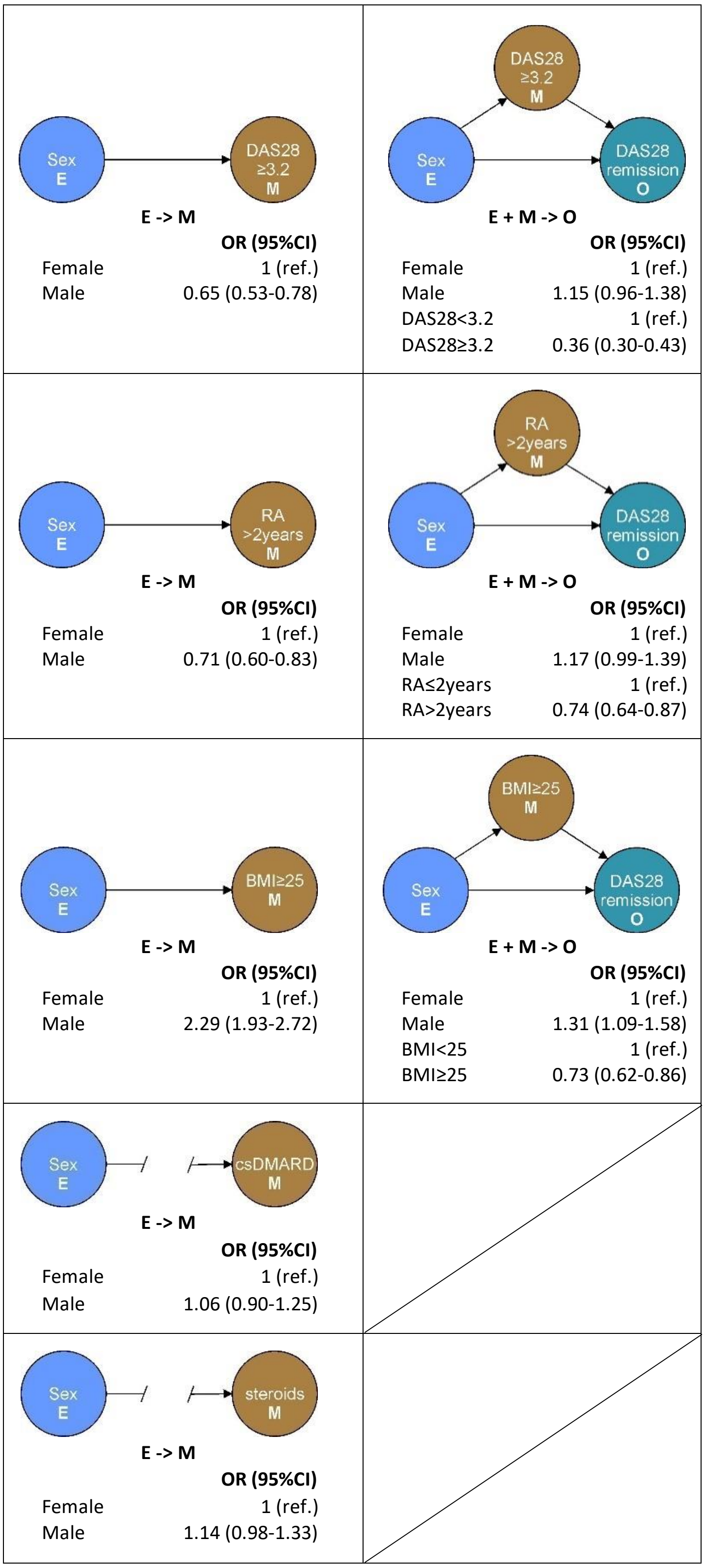

S5 Fig. Mediation analyses. Directed acyclic graphs (DAGs) showing the dependencies between the study exposure sex/gender (E; blue balloons), the study outcome DAS28-remission (O; green balloons), and the potential mediators (M; brown balloons), for the association between sex/gender and DAS28-remission. Each DAG is accompanied by the respective odds ratio (OR) with 95% confidence interval (CI). Abbreviations: DAS28 Disease Activity Score 28; RA rheumatoid arthritis; BMI body mass index; csDMARD conventional synthetic disease-modifying anti-rheumatic drug; E exposure; O outcome; M mediator.
