# Supplementary material for: Lower odds of remission among women with rheumatoid arthritis: A cohort study in the Swiss Clinical Quality Management cohort
Source: PLoS One. 2022 Oct 20;17(10):e0275026. doi: 10.1371/journal.pone.0275026 (PMC9584448; doi:10.1371/journal.pone.0275026)
Supplement: S1 Table — The 41.2% of the patients had complete information on every variable included in the MICE. (PDF) [file pone.0275026.s004.pdf]

S2 Table. Details about the variables and methods used in the multiple imputations by chain equations (MICE). The 41.2% of the patients had complete information on every variable included in the MICE.

Variable	Predicted	Used as predictor	Method	Missingness	Levels
Sex	-	yes	-	0%	women; men
BMI	yes	yes	pmm	16.3%	-
Age at index	-	yes	-	0%	-
RA disease duration	yes	yes	pmm	3.4%	-
Smoker ever before	-	yes	-	0%	yes; no
b/tsDMARDs	-	yes	-	0%	TNFi; other biologic; tsDMARD
csDMARD use	-	yes	-	0%	yes; no
Glucocorticoid use	-	yes	-	0%	yes; no
RF	yes	yes	logreg	4.3%	yes; no
Anti-CCP	yes	yes	logreg	26.7%	yes; no
Seropositivity	yes	yes[a]	logreg	9.9%	yes; no
ESR	yes	yes	pmm	12.1%	-
SJC28	yes	yes	pmm	1.7%	-
TJC28	yes	yes	pmm	2.0%	-
RADAI-5	yes	yes	pmm	22.6%	-
DAS28-ESR	yes	yes[b]	pmm	13.3%	-
Outcome	-	yes	-	0%	yes; no

[a] Excluded as predictor in models for RF and anti-CCP.

[b] Excluded as predictor in for SJC28, TJC28 and ESR.

Abbreviations: BMI body mass index; b/tsDMARD biologic or targeted synthetic disease modifying anti-rheumatic drug; TNF tumour necrosis factor; csDMARD conventional synthetic disease modifying anti-rheumatic drug; RF rheumatoid factor; Anti-CCP anti-cyclic citrullinated peptide antibodies; ESR erythrocyte sedimentation rate; SJC28 number of swollen joint counts counting 28; TJC28 number of tender joint counts counting 28; RADAI-5 Rheumatoid Arthritis Disease Activity Index-5; DAS28 Disease Activity Score 28; logreg logistic regression; pmm predictive mean matching.
