# Supplementary material for: Lower odds of remission among women with rheumatoid arthritis: A cohort study in the Swiss Clinical Quality Management cohort
Source: PLoS One. 2022 Oct 20;17(10):e0275026. doi: 10.1371/journal.pone.0275026 (PMC9584448; doi:10.1371/journal.pone.0275026)
Supplement: S2 Table — Logistic regression assessing the effect of sex/gender on the study outcomes, with maximum follow-up of 12-months. (PDF) [file pone.0275026.s005.pdf]

S6 Table. Sensitivity analysis, excluding patients without any record on the outcome information during follow-up. Logistic regression assessing the effect of sex/gender on the study outcomes, with maximum follow-up of 12-months.

Sensitivity analysis<br>Outcome at ≤12-months	n sample size	n events	OR (95% CI)	ORadj (95% CI)
**DAS28-remission**	2464	1033		
Women		762	1 (ref.)	1 (ref.)
Men		271	1.36 (1.12-1.64)	1.39 (1.15-1.68)
**DAS28-rem/LDA**	2464	1453		
Women		1096	1 (ref.)	1 (ref.)
Men		357	1.23 (1.02-1.50)	1.25 (1.03-1.52)
**RADAI-5-remission**	2169	544		
Women		413	1 (ref.)	1 (ref.)
Men		131	1.06 (0.85-1.34)	1.09 (0.87-1.37)

Abbreviations: n number; OR odds ratio; ORadj odds ratio adjusted for age and seropositivity; DAS28 Disease Activity Score 28; rem/LDA remission or low disease activity; RADAI-5 Rheumatoid Arthritis Disease Activity Index-5.
