# Supplementary material for: Lower odds of remission among women with rheumatoid arthritis: A cohort study in the Swiss Clinical Quality Management cohort
Source: PLoS One. 2022 Oct 20;17(10):e0275026. doi: 10.1371/journal.pone.0275026 (PMC9584448; doi:10.1371/journal.pone.0275026)
Supplement: S3 Table — Changes in individual clinical endpoints and composite disease activity scores (delta: Follow-up value—baseline). As a note, median values below zero reflect improvement, reduction of the scores. (PDF) [file pone.0275026.s006.pdf]

S7 Table. Post-hoc analysis. Changes in individual clinical endpoints and composite disease activity scores (delta: follow-up value - baseline). As a note, median values below zero reflect improvement, reduction of the scores.

	**Women (n=2839)**	**Men (n=883)**	**p value**
ΔESR (median [IQR])	-4.00 [-14.00, 0.00]	-5.00 [-17.00, 0.00]	0.226
missing	1220 (42.97%)	386 (43.71%)	
ΔCRP (median [IQR])	-0.20 [-0.90, 0.00]	-0.30 [-1.40, 0.00]	**0.030**
missing	2039 (71.82%)	612 (69.31%)	
ΔTJC28 (median [IQR])	-3.00 [-7.00, 0.00]	-2.00 [-6.00, 0.00]	0.228
missing	908 (31.98%)	311 (35.22%)	
ΔSJC28 (median [IQR])	-3.00 [-7.00, 0.00]	-3.00 [-7.00, 0.00]	0.727
missing	903 (31.81%)	306 (34.65%)	
ΔDAS28-ESR (median [IQR])	-1.30 [-2.20, -0.30]	-1.20 [-2.40, -0.40]	0.745
missing	1258 (44.31%)	404 (45.75%)	
ΔDAS28-CRP (median [IQR])	-1.45 [-2.20, -0.60]	-1.40 [-2.30, -0.50]	0.720
missing	2073 (73.02%)	633 (71.69%)	
ΔRADAI5 (median [IQR])	-1.40 [-3.00, -0.20]	-1.40 [-3.00, -0.20]	0.997
missing	1522 (53.61%)	471 (53.34%)	

Abbreviations: IQR interquartile range; ESR erythrocyte sedimentation rate; CRP C-reactive protein; TJC28 tender joint count 28; SJC28 swollen joint count; DAS28 disease activity score 28; RADAI-5 rheumatoid arthritis disease activity index-5
