# Supplementary material for: Lower odds of remission among women with rheumatoid arthritis: A cohort study in the Swiss Clinical Quality Management cohort
Source: PLoS One. 2022 Oct 20;17(10):e0275026. doi: 10.1371/journal.pone.0275026 (PMC9584448; doi:10.1371/journal.pone.0275026)
Supplement: S1 Equations — (PDF) [file pone.0275026.s007.pdf]

S1 Equations

$$DAS28_{ESR} = (0.56 \times \sqrt{tjc28} + 0.28 \times \sqrt{sjc28} + 0.7 \times ln(ESR)) \times 1.08 + 0.16 \quad \textbf{(1)}$$

$$DAS28_{CRP} = \left(0.56 \times \sqrt{tjc28} + 0.28 \times \sqrt{sjc28} + 0.36 \times ln(CRP + 1)\right) \times 1.10 + 1.15 \quad \textbf{(2)}$$

Abbreviations: DAS28 Disease Activity Score 28; tjc28 Number of tender joints, counting 28; sjc28 Number of swollen joints, counting 28; ESR Erythrocyte sedimentation rate (mm/h); CRP C-reactive protein (mg/dL).
